# Posicionamiento de Ecuador en la agenda de salud global como producto de la reforma sectorial

**DOI:** 10.26633/RPSP.2017.55

**Published:** 2017-05-15

**Authors:** Cristina Luna, Carlos Andrés Emanuele, Daniel De La Torre

**Affiliations:** 1 Consultora independiente Consultora independiente Quito Ecuador Consultora independiente, Quito, Ecuador; 2 Organización Panamericana de la Salud United States of America. Organización Panamericana de la Salud Washington, D.C United States of America Organización Panamericana de la Salud, Washington, D.C, United States of America; 3 Ministerio de Salud Póblica del Ecuador Ministerio de Salud Póblica del Ecuador Quito Ecuador Ministerio de Salud Póblica del Ecuador, Quito, Ecuador.

**Keywords:** Reforma del sector salud, salud global, relaciones internacionales, diplomacia, Ecuador, Health care reform, global health, internationality, diplomacy, Ecuador

## Abstract

**Objetivo.:**

Analizar las estrategias implementadas por el Ministerio de Salud Pública del Ecuador (MSP) para posicionar al país en la agenda de salud global durante el periodo 2011-2015 como producto del proceso de reforma del sector salud.

**Método.:**

Revisión documental y entrevistas con actores clave de instancias nacionales e internacionales respecto al posicionamiento en el ámbito de la salud global durante el periodo estudiado.

**Resultados.:**

Se observa que el proceso de reforma generó un nuevo marco para la gestión de las relaciones internacionales en materia de salud. El MSP implementó estrategias y mecanismos para situar las prioridades e intereses nacionales en salud en la agenda de salud global a escala bilateral, regional y mundial. Como consecuencia de ello, el país asumió un papel de liderazgo en ciertos procesos y alcanzó el reconocimiento en diversos foros internacionales.

**Conclusiones.:**

El proceso de reforma del MSP contribuyó al reconocimiento y al establecimiento de las prioridades de política pública de Ecuador dentro de la agenda de salud global a través de estrategias tales como otorgar importancia a la proyección de las prioridades nacionales a nivel de la salud global, liderar el abordaje de la salud global ejercido por la Máxima Autoridad Sanitaria, desarrollar capacidades técnicas y destrezas en la Oficina de Relaciones internacionales, y lograr la concienciación en las instancias técnicas.

## Abstract (en)

**Objective.:**

Analyze strategies implemented by Ecuador’s Ministry of Public Health (MPH) to position the country in the global health agenda during the period 2011-2015 as a result of health sector reform.

**Method.:**

Documentary review and interviews with stakeholders in national and international agencies with respect to positioning in the global health sphere during the study period.

**Results.:**

It was observed that the reform process produced a new framework to manage international health relations. The MPH implemented strategies and mechanisms to place national health priorities and interests on the global health agenda at bilateral, regional, and global levels. As a result, the country took a leadership role in certain processes and attained recognition at various international forums.

**Conclusions.:**

The MPH reform process contributed to recognition and establishment of Ecuador’s public policy priorities in the global health agenda through strategies such as giving importance to putting national priorities on the global health agenda, guiding the global health approach exercised by the highest health authority, developing technical capabilities and skills in the International Relations Office, and raising awareness in technical bodies.

Algunos desafíos relevantes de la reforma del sector salud en Ecuador son la sostenibilidad del progreso logrado, la continuidad a la institucionalización de los procesos y estrategias desarrollados, y el fortalecimiento de la coordinación intersectorial en lo que atañe a la política exterior y la diplomacia.

Ecuador, a partir de la aprobación de la Constitución en 2008, llevó a cabo un proceso de reforma profunda, que planteó un nuevo escenario para la gestión de las relaciones internacionales en materia de salud en el país. En este contexto, el Ministerio de Salud Pública (MSP) implementó procesos de reestructuración institucional a fin de disponer de los recursos necesarios para responder al nuevo marco normativo.

En este marco, el MSP, conforme a su mandato de fortalecer su papel rector como Autoridad Sanitaria Nacional, y en virtud de los preceptos de la política exterior del Gobierno Nacional y la creciente relevancia de la salud en la agenda internacional, adoptó un conjunto de medidas estratégicas para situar al país en materia sanitaria a nivel mundial, regional y bilateral.

En consecuencia, resulta de gran interés identificar y describir las estrategias y los mecanismos desarrollados, gracias a los cuales fue posible lograr el reconocimiento internacional de los intereses y prioridades del país en materia de salud, para poder destacar los aspectos clave que permitan replicarlos e institucionalizarlos, dado que no existe documentación al respecto.

## MATERIALES Y MÉTODOS

Los métodos empleados para elaborar este artículo fueron una revisión documental y entrevistas con actores clave de instancias nacionales e internacionales vinculadas con la agenda de salud global respecto al posicionamiento del Ecuador a escala internacional durante el periodo en estudio.

Se realizó una revisión principalmente de las normativas (Constitución, Plan Nacional del Buen Vivir), documentación institucional (Estatuto, Oficios y Memorandos), boletines de prensa y documentación de organismos internacionales, centrando la bús-queda en temas de salud y relaciones internacionales. En total, se seleccionaron 27 fuentes bibliográficas entre fuentes primarias, fuentes secundarias y entrevistas.

Para realizar las entrevistas, y con el fin de tener una perspectiva tanto nacional como internacional, se eligieron tres representantes que desempeñaron un papel importante en temas de salud global: Carina Vance Mafla, Ministra de Salud Pública del Ecuador (2012-2015) y actual Directora Ejecutiva del Instituto Suramericano de Gobierno en Salud, María Verónica Espinosa Serrano, Ministra de Salud Pública del Ecuador, y María Cecilia Acuña, Asesora de Sistemas y Servicios de Salud de la OPS/OMS en México.

## RESULTADOS

### Prioridades de política exterior para la agenda de salud global

En 2008, Ecuador aprobó una nueva Constitución de la República, la cual, en materia sanitaria, destaca el posicionamiento de la salud como un derecho que debe ser garantizado por el Estado de forma universal, equitativa, gratuita y sin discriminación y que, a su vez, se vincula con el ejercicio de otros derechos que sustentan el Buen Vivir: *“Art. 32.- La salud es un derecho que garantiza el Estado, cuya realización se vincula al ejercicio de otros derechos /…/. La prestación de los servicios de salud se regirá por los principios de equidad, universalidad, solidaridad, interculturalidad, calidad, eficiencia, eficacia, precaución y bioética, con enfoque de género y generacional”* (1).

A este artículo se suman una serie de mandatos que viabilizan el efectivo ejercicio del derecho a la salud. La Carta Magna establece la importancia de salvaguardar el bienestar sobre el capital. En su Artículo 363 se señala: *“/…/. En el acceso a medicamentos, los intereses de la salud pública prevalecerán sobre los económicos y comerciales”* (1).

En un contexto de coherencia, los principios instaurados a nivel nacional fueron también extrapolados a los lineamientos que marcan la política exterior. El Artículo 421 establece que *“La aplicación de los instrumentos comerciales internacionales no menoscabará, directa o indirectamente, el derecho a la salud, el acceso a medicamentos, insumos, servicios, ni los avances científicos y tecnológicos”* (1).

Por su parte, en el Artículo 423 de la Constitución se estipula que la integración, particularmente con los países de Latinoamérica y del Caribe, es un objetivo estratégico, para lo cual se establece como objetivo fortalecer la articulación de las legislaciones nacionales haciendo hincapié en los derechos y regímenes laboral, migratorio, fronterizo, ambiental, social, educativo, cultural y****de****salud pública (1). Por otro lado, en concordancia con los preceptos constitucionales en materia de relaciones internacionales, el Plan Nacional del Buen Vivir plantea en su Objetivo 12 “*Garantizar la soberanía y la paz, profundizar la inserción estratégica en el mundo y la integración latinoamericana*” (2).

El Plan vigente propone doce objetivos y en el tercero de ellos se establece “*Mejorar la calidad de vida de la población*” a través del fortalecimiento de políticas intersectoriales y de la consolidación del Sistema Nacional de Inclusión y Equidad Social. Para su consecución, se propone entre otros propósitos, mejorar la calidad y calidez de los servicios sociales de atención, y garantizar la salud de la población desde la generación de un ambiente y prácticas saludables (2).

### Alineación con la agenda de desarrollo global

Las metas del Plan Nacional para el Buen Vivir trascienden aquellas fijadas por las Naciones Unidas a través de los Objetivos de Desarrollo del Milenio (ODM) y se constituyen en un referente internacional por medio de profundas transformaciones y resultados concretos (2).

En 2007, el Gobierno ratificó los ODM. Sin embargo, se han cuestionado sus estándares, toda vez que desde la perspectiva ecuatoriana son limitados y están enfocados a un cumplimiento mínimo, no responden a la realidad de los países (especialmente de los menos desarrollados) y no se dirigen a cambios reales encaminados a la reducción de brechas sociales y la redistribución de la riqueza sobre la base del modelo de desarrollo planteado por Ecuador (2). De las 21 metas establecidas en los ocho ODM fijados Ecuador alcanzó 20 y, la pendiente, la mortalidad materna, continúa constituyendo un reto (3).

### Ecuador en la Gobernanza de Salud Global

Ecuador fue elegido miembro del Consejo Ejecutivo de la Organización Mundial de la Salud (OMS) desde 2011 hasta 2013, lo cual marcó el inicio de un periodo en el cual se le dio particular realce y prioridad a la agenda de salud global. Desde entonces, el país se ha posicionado en varios espacios importantes de la salud global, entre los cuales cabe destacar la elección de Ecuador como miembro del Comité Ejecutivo de la OPS (2013-2016) (4), la designación como Presidente del 52º Consejo Directivo de la OPS (2013-2014), la elección como Miembro del Subcomité de Programa, Presupuesto y Administración del Comité Ejecutivo de la OPS (2015-2016), la designación como Presidente del Comité Ejecutivo de la OPS (2015-2016) (5), la elección como Vicepresidente del Grupo Asesor del Plan Estratégico de la OPS (2014-2015), y su selección como miembro en el Grupo de Trabajo para el Plan Estratégico de la OPS, 2013-2014.

Este reconocimiento se extiende también a otros organismos internacionales como ONUSIDA, donde Ecuador fue elegido en 2015 Relator de la Junta Coordinadora ONUSIDA para el periodo 2016-2018. Estos logros se suman al conseguido en 2012 cuando se celebró la Conferencia Regional sobre Población y Desarrollo de América Latina y el Caribe, en la cual Ecuador fue país anfitrión y Presidente. Además, Ecuador ha proyectado temas de prioridad nacional en la agenda de salud subregional en el seno del Consejo de Salud Suramericano de la Unión de Naciones Suramericanas, así como en el del Organismo Andino de Salud-Convenio Hipólito Unanue.

La presencia del Ecuador en estos espacios ha permitido fijar las prioridades nacionales e internacionales de salud global y, a su vez, dotar a la política nacional del respaldo de bloques subregionales y regionales.

### Estrategias de posicionamiento

En el marco de la reforma estructural del Estado, el MSP también entró en un proceso de reestructuración, dado que los cambios planteados en el nuevo orden de gestión pública le otorgaron mayores competencias, principalmente la recuperación del papel rector (6). Es así como en 2011 se planteó una nueva estructura organizativa del MSP en la cual se modificó la oficina de cooperación internacional, la “Coordinación de Cooperación Nacional e Internacional”, que estaba subordinada a la Subsecretaría de Planificación como un subproceso y pasó a ser una unidad de la asesoría directamente dependiente de la Máxima Autoridad de Salud Pública. De este modo, se convirtió en la “Dirección Nacional de Cooperación y Relaciones Internacionales” (7).

Dicho proceso requirió un cambio organizativo profundo, que demandó el fortalecimiento de la gestión de las relaciones internacionales en salud. Al depender de manera directa del Despacho Ministerial, la Dirección asumió mayores responsabilidades, lo cual resultó en una mayor priorización de los temas de salud global (6).

En este sentido, la reforma de la Oficina le otorgó responsabilidades, atribuciones y productos correspondientes al nuevo estatus de Dirección, involucrando así facultades relacionadas con la asesoría, la negociación y la gestión de la cooperación internacional, para “asegurar la adecuada participación del Ministerio de Salud Pública en instancias y acuerdos bilaterales y multilaterales, armonizando los lineamientos de la Política Exterior con las prioridades establecidas en el sector salud; que permitan complementar las acciones para la consecución de los objetivos planteados en los Planes de Desarrollo del País”** (8).

Durante el proceso de reestructuración, se elaboraron manuales de procesos y manuales de puestos, lo cual incluyó la incorporación de personal con formación académica y experiencia laboral conforme a las atribuciones y responsabilidades otorgadas. Como consecuencia, la Dirección fue dotándose progresivamente de recursos humanos con perfiles óptimos, lo que favorece el cumplimiento de lo estipulado en el nuevo Estatuto (9, 10).

A partir de 2012, en reconocimiento del posicionamiento y de la importancia otorgada a la Dirección, la Ministra de Salud Pública suscribió disposiciones dirigidas tanto a las unidades internas del MSP como a cooperantes internacionales, en las cuales se establece que todas las gestiones de la cooperación y relaciones internacionales de esa Cartera de Estado sean coordinadas y canalizadas a través de la Dirección Nacional de Cooperación y Relaciones Internacionales, todo ello con el fin de garantizar que guarden concordancia con las políticas, los objetivos y el estatuto del MSP y de los lineamientos de política exterior del Ecuador (11-14).

El resultado de todo ello fue la creación de una oficina de relaciones internacionales de alto nivel institucional, logro que constituye una consolidación integral del posicionamiento ante los cuerpos directivos de organismos multilaterales, así como de estrategias de cooperación a nivel bilateral. Desde la dirección de Cooperación y Relaciones Internacionales los temas se analizaron de manera más directa y crítica y otorgando un valor agregado a las aportaciones de las unidades técnicas del MSP desde la visión de la salud internacional y la diplomacia sanitaria (10).

Por otro lado, partiendo de los preceptos de la política exterior del Gobierno Nacional y de la creciente relevancia de la salud en la agenda internacional, especialmente en los procesos de consolidación de los ODS, se estrechó la comunicación y la relación institucional entre el MSP y el Ministerio de Relaciones Exteriores y Movilidad Humana, lo cual ha favorecido la posición de Ecuador en materia de salud a escala internacional. Del mismo modo, y en virtud de la preponderancia de las políticas sociales, la coordinación y las interrelaciones con el Ministerio Coordinador de Desarrollo Social también han desempeñado un papel clave para la consolidación de las estrategias de posicionamiento internacional en materia sanitaria (10).

### Incidencia en la agenda de salud global

Los preceptos de la Constitución del Ecuador de 2008 y del Plan Nacional de Buen Vivir en materia de relaciones internacionales y de la salud pública marcaron la dirección de las acciones requeridas para situar a Ecuador en el plano internacional y constituyeron un pilar para contar con un respaldo jurídico y regulatorio en el marco del efectivo ejercicio del derecho a la salud, así como en la construcción de un país equitativo, inclusivo y saludable (15).

La reforma institucional del MSP y el conjunto de medidas estratégicas adoptadas potenciaron la gestión internacional, situaron a Ecuador como un actor proactivo, crítico y colaborador en temas de salud global, y lo convirtieron en un líder de referencia en salud de la comunidad internacional (15).

### Promoción y adopción del Plan de Acción Mundial de la OMS sobre discapacidad 2014-2021

En 2013, al amparo de la acción que el Gobierno Nacional realizó en favor de la inclusión social, equidad y bienestar de las personas con discapacidad, Ecuador incluyó un punto en la agenda del 132º Consejo Ejecutivo de la OMS y presentó una propuesta de resolución ante el pleno. En mayo de 2013, la 66ª Asamblea Mundial de la Salud aprobó la Resolución WHA66.9 referente a la discapacidad, en la cual se solicitaba a la OMS que apoyara a los países para que sigan las recomendaciones del Informe Mundial sobre Discapacidad 2011. Sobre la base de la iniciativa ecuatoriana, se solicitó a la Secretaría de la OMS preparar un plan de acción integral con resultados medibles en el plazo de un año (16).

En cumplimiento de dicha resolución, el 5 y 6 de noviembre se realizó en Quito, Ecuador, la Consulta Regional en las Américas sobre el Plan de Acción 2014-2021 sobre discapacidades de la OMS (17). Finalmente: “En una decisión histórica, la 67.ª Asamblea Mundial de la Salud adoptó una resolución por la que ratificaba el Plan de acción mundial de la OMS sobre discapacidad 2014-2021: mejor salud para todas las personas con discapacidad. El Plan de acción dará un impulso considerable a los esfuerzos de la OMS y los gobiernos por mejorar la calidad de vida de mil millones de personas con discapacidad de todo el mundo” (18).

Para subrayar la importancia de esta prioridad nacional de alcance global, es necesario destacar el origen de esta iniciativa en el seno del Organismo Andino de Salud con la “Política Andina en Salud para la Prevención de la Discapacidad y Para la Atención, Habilitación/Rehabilitación Integral de las Personas con Discapacidad”, que se convirtió en prioridad de los países de la Unión de Naciones Suramericanas, que presentaron el tema en la Asamblea Mundial de Salud para su aprobación a nivel global.

### Estrategia de Acceso Universal a la Salud y Cobertura Universal de Salud

Cabe destacar asimismo la participación activa de la delegación ecuatoriana en la construcción de la Estrategia para el Acceso Universal a la Salud y la Cobertura Universal de Salud en 2014. Durante las negociaciones y los debates en los Cuerpos Directivos de la OPS, se logró por primera vez en la historia de la Organización la adopción de un lenguaje que incluye el derecho a la salud. Esto no se ha conseguido aún en la OMS y sigue siendo un reto pendiente. Asimismo, se aprobaron recomendaciones para alcanzar en los países el acceso y la cobertura universal en salud (19).

### Plan de Acción para la Prevención de la Obesidad en la Niñez y la Adolescencia

La agenda del 53º Consejo Ejecutivo de la OPS incluyó temas relacionados con el desarrollo sanitario en la Región de las Américas, entre ellos el “Plan de Acción para la Prevención de la Obesidad en la Niñez y la Adolescencia”. En el documento publicado, se proyectan experiencias de política pública adoptadas a nivel nacional para la consideración y adopción por parte de todos los países de la Región. En él se destacan dos líneas principales de acción relacionadas, primero, con la necesidad de adoptar un esquema de etiquetado de alimentos, que, a su vez, tenga incidencia en el dispendio de alimentos en los bares escolares, y, segundo, con la importancia del trabajo intersectorial, que incluye la reglamentación de la actividad física diaria en los establecimientos educativos. Para dicho Plan, las experiencias nacionales de Ecuador contribuyeron en la construcción de sus objetivos, indicadores y metas (20).

### Experiencia del reglamento de etiquetado de alimentos procesados

Un aspecto que ha sido fundamental para el posicionamiento internacional de Ecuador es el reconocimiento de normativas exitosas en política pública del país. La experiencia en la construcción, negociación y promulgación nacional en 2014 del Reglamento Sanitario de Etiquetado de Alimentos Procesados para el Consumo Humano sitúa al país como el primero en el mundo en poseer un etiquetado gráfico obligatorio en los alimentos procesados (21).

Como consecuencia de ello, las estrategias para reafirmar prioridades nacionales e internacionales se orientan al posicionamiento y reconocimiento del Reglamento como experiencia exitosa en el contexto de la promoción de la alimentación saludable y en cumplimiento del objetivo de mejorar la calidad de vida de todos los ciudadanos (22).

### Acuerdos binacionales y desarrollo de proyectos en la zona de frontera con Colombia y Perú

Entre los logros alcanzados destaca también el trabajo binacional, particularmente con Colombia y Perú, de los cuales parte una voluntad política, emanada directamente desde una directriz presidencial (Encuentros Presidenciales), y la celebración de reuniones de coordinación y supervisión de Encuentros Presidenciales y Sectoriales. La articulación binacional busca mejorar la calidad de vida de los ciudadanos y concibe la salud como eje integrador de los países y herramienta para el desarrollo de las zonas fronterizas gracias a la planificación conjunta a corto, mediano y largo plazo, conjugando prioridades y problemas comunes, y buscando soluciones sistemáticas, integrales y mancomunadas a través de planes quinquenales de acción.

Merced al acuerdo de atención recíproca entre Ecuador y Perú (23), los ciudadanos peruanos tienen acceso universal y gratuito a la salud en territorio ecuatoriano y los ciudadanos ecuatorianos tienen acceso gratuito en territorio peruano a las prestaciones incluidas en el Plan Esencial de Aseguramiento en Salud (24). Estas iniciativas se acompañan de un trabajo binacional conjunto en temas de promoción de la salud y enfermedades transmitidas por vectores (25).

El trabajo con Colombia apunta a la implementación de un sistema binacional de atención en salud mediante el cual los ciudadanos puedan acceder a los servicios de salud. Esta iniciativa comprende una serie de aspectos complementarios, como un sistema de vigilancia epidemiológico conjunto, un sistema de registro de atenciones compartido, una cartera de servicios homologada y de trabajo articulado, y acciones en las esferas de la salud sexual y reproductiva, la salud infantil y las enfermedades transmitidas por vectores (26, 27).

## DISCUSIÓN

El proceso de reforma del MSP, además de alcanzar logros nacionales mediante el fortalecimiento de la rectoría de la Autoridad Sanitaria, contribuyó al reconocimiento y posicionamiento de las prioridades de política pública de Ecuador dentro de la agenda de salud global.

De lo anteriormente expuesto se puede inferir que existen varios factores, de carácter interno, que han favorecido el posicionamiento del país en el ámbito de la salud global, que todos ellos nacen de la reforma institucional del MSP y que, a su vez, permitieron poner en práctica una serie de estrategias como (i) establecer la importancia en la proyección de las prioridades nacionales en la agenda de salud global, (ii) liderar a nivel nacional el abordaje de la agenda de salud global ejercido por la Máxima Autoridad Sanitaria, y (iii) desarrollar las capacidades técnicas y destrezas en la Oficina de Relaciones Internacionales y logar la concienciación en las instancias técnicas.

También es necesario hacer alusión a factores exógenos que han incidido en la proyección de prioridades políticas durante el periodo en análisis, y especialmente a la existencia de una coyuntura regional, con países con visiones y objetivos comunes cuya actuación parte de un enfoque de derechos en el cual el derecho a la salud tiene uno de los primeros órdenes de prelación. Esta coyuntura regional vio el surgimiento de bloques regionales como la Unión de Naciones Suramericanas, que ha tenido una incidencia marcada tanto en la agenda de salud subregional, como en la agenda de salud global. Asimismo, se pueden mencionar las actuaciones de otros bloques subregionales que han avanzado en materia sanitaria dentro del Organismo Andino de Salud y el Mercado Común del Sur.

Si bien podría considerarse positiva la evolución de la gestión de la agenda de salud global en Ecuador, quedan todavía varios desafíos pendientes. En primera instancia, aún es necesario fortalecer la participación en foros internacionales de delegaciones fortalecidas con los recursos necesarios y disponibles, tanto financieros como humanos. Esto supone actuar en un ámbito operacional circunscrito a temas considerados altamente estratégicos, mediante la priorización eficiente respecto de otros temas.

Es importante mencionar que Ecuador ha avanzado considerablemente en la articulación de política pública intersectorial gracias a los Ministerios Coordinadores. A pesar de estos avances, es indispensable adoptar un abordaje intersectorial de la salud, con la participación de otras instituciones tanto en espacios internacionales sanitarios, como en otros foros de deliberación donde se abordan asuntos que influyen directa o indirectamente en el sector. Se reconoce que este es un desafío al que se enfrentan muchos otros países, que es de suma relevancia dada la creciente importancia de la salud global en la agenda nacional, regional e internacional, y que su inserción en las agendas es cada vez más variada en cuanto a los organismos que la abordan.

Finalmente, el éxito de la reforma en relación con la gestión de las relaciones internacionales en materia de salud vendrá determinado por la sostenibilidad del progreso conseguido hasta ahora. Para ello, es importante continuar con la institucionalización de los procesos y las estrategias, tanto en la Oficina de Relaciones Internacionales, como en las instancias técnicas. De este modo, será posible asegurar que Ecuador continúe desempeñando un papel importante en la agenda de salud global en función de sus prioridades nacionales e internacionales en materia de salud.

## Agradecimientos.

Los autores agradecen a Verónica Espinosa (MSP, Ecuador) y a Cecilia Acuña (OPS, México) su ayuda en la redacción de este manuscrito, así como a todos aquellos que contribuyeron al posicionamiento del Ecuador, empezando por los Representantes de la OPS/OMS en Ecuador, Dres. Mario Valcárcel, Manuel Peña, y Gina Tambini, y su equipo en la Representación, y a todos los países hermanos de la Región de las Américas, particularmente a los Estados Miembros de UNASUR, con los cuales se propició una coyuntura que permitió avanzar en la mejora de la salud con un enfoque de derechos. Finalmente, expresan su agradecimiento a Carina Vance, Ministra de Salud Pública, que lideró este proceso, por su apoyo en la redacción de este artículo y por habernos dado la oportunidad de ser parte de este cambio.

## Declaración.

Las opiniones expresadas por los autores son de su exclusiva responsabilidad y no reflejan necesariamente los criterios ni la política de la *RPSP/PAJPH* o de la OPS.
